# O-46 - TUBERCULOSIS INCIDENTS IN HEALTH AND SOCIAL CARE SETTINGS IN THE SOUTH EAST OF ENGLAND 2018 - 2024

**DOI:** 10.1093/pubmed/fdag046.041

**Published:** 2026-07-09

**Authors:** Ms Clare Sawyer, Ms Clare Humphreys, Ms Claire Winslade, Dr Flora Kaminski, Ms Grace King, Ms Charlotte Anderson

**Affiliations:** Field Services South East and London, UK Health Security Agency; South East Health Protection Team, UK Health Security Agency; South East Health Protection Team, UK Health Security Agency; South East Health Protection Team, UK Health Security Agency; Field Services South East and London, UK Health Security Agency; Field Services South East and London, UK Health Security Agency

## Abstract

**Background:**

Pulmonary Tuberculosis (TB) in health and social care workers (HSCWs) leads to risk of transmission to vulnerable patients. Guidelines recommend HSCWs be free from TB disease, and immunised where appropriate.

**Objectives:**

We describe TB cases and incidents related to HSCWs, and settings, in the South East of England, between 2022 - 2024.

**Methods:**

We describe TB cases resident in the South East with “health and social care” as their occupation, notified to National TB Surveillance (NTBS) between 2018 and 2024. We describe incidents involving TB in a staff member in social care setting (residential/nursing home) reported to UKHSA

**Results:**

The proportion of individuals with TB in the South East who were HSCWs increased from 10% in 2018 (46/425) to 18% by 2024 (100/460). Among HSCWs with TB, the proportion born outside the UK increased from 78% in 2018 (36/46) to 99% in 2024 (99/100). In 2024, 59% were notified less than 5 years since arriving in the UK. 22 incidents were identified, mainly in care homes (15/22). The median duration of time spent in the setting whilst infectious was 15 weeks (range 0.7 to 52 weeks). Screening of contacts was undertaken in 63% (14/22) of incidents. The median number of contacts identified was 8 (range: 4-85) and the median number screened was 4 (1-73). For three incidents, further cases were identified. Barriers to screening included capacity to consent, and practical difficulties in arranging appropriate screening due to mobility limitations.

**Conclusions:**

We recommend improved occupational health provision for Social Care Workers, in line with NHS HCWs, particularly for those from high incidence TB countries, including pre-employment screening, symptom awareness and assessment to reduce prolonged infectious exposure in social care settings and prevent further cases.

